# NTproBNP in insulin-resistance mediated conditions: overweight/obesity, metabolic syndrome and diabetes. The population-based Casale Monferrato Study

**DOI:** 10.1186/s12933-017-0601-z

**Published:** 2017-09-25

**Authors:** Stefano Baldassarre, Salvatore Fragapani, Antonio Panero, Debora Fedele, Silvia Pinach, Manuela Lucchiari, Anna Rita Vitale, Giulio Mengozzi, Gabriella Gruden, Graziella Bruno

**Affiliations:** 1 Dept. of Medical Sciences, University of Torino, corso Dogliotti 14, 10126 Turin, Italy; 2 Clinical Chemistry Laboratory, Azienda Ospedaliero-Universitaria Città della Salute e della Scienza, Turin, Italy

**Keywords:** Natriuretic peptides, Metabolic syndrome, CRP, CVD, Survey

## Abstract

**Background and aims:**

NTproBNP and BNP levels are reduced in obese subjects, but population-based data comparing the pattern of this relationship in the full spectrum of insulin-resistance mediated conditions, overweight/obesity, metabolic syndrome and diabetes, are limited.

**Methods:**

The study-base were 3244 individuals aged 45–74 years, none of whom had heart failure, 1880 without diabetes and 1364 with diabetes, identified as part of two surveys of the population-based Casale Monferrato Study. All measurements were centralized. We examined with multiple linear regression and cubic regression splines the relationship between NTproBNP and BMI, independently of known risk factors and confounders. A logistic regression analysis was also performed to assess the effect of overweight/obesity (BMI ≥ 25 kg/m^2^), diabetes and metabolic syndrome on NTproBNP values.

**Results:**

Out of the overall cohort of 3244 people, overweight/obesity was observed in 1118 (59.4%) non-diabetic and 917 (67.2%) diabetic subjects, respectively. In logistic regression, compared to normal weight individuals, those with a BMI ≥ 25 kg/m^2^ had a OR of 0.70 (95% CI 0.56–0.87) of having high NTproBNP values, independently of diabetes. As interaction between diabetes and NTproBNP was evident (p < 0.001), stratified analyses were performed. Diabetes either alone or combined with overweight/obesity or metabolic syndrome enhanced fourfold and over the OR of having high NTproBNP levels, while the presence of metabolic syndrome alone had a more modest effect (OR 1.54, 1.18–2.01) even after having excluded individuals with CVD. In the non-diabetic cohort, obesity/overweight and HOMA-IR ≥ 2.0 decreased to a similar extent the ORs of high NTproBNP [0.76 (0.60–0.95) and 0.74 (0.59–0.93)], but the association between overweight/obesity and NTproBNP was no longer significant after the inclusion into the model of HOMA-IR, whereas CRP > 3 mg/dl conferred a fully adjusted OR of 0.65 (0.49–0.86).

**Conclusions:**

NT-proBNP levels are lower in overweight/obesity, even in those with diabetes. Both insulin-resistance and chronic low-grade inflammation are involved in this relationship. Further intervention studies are required to clarify the potential role of drugs affecting the natriuretic peptides system on body weight and risk of diabetes.

## Background

Epidemiological studies have pointed out that obese individuals have lower plasma levels of natriuretic peptides than those with normal weight, despite the higher prevalence of hypertension and left ventricular hypertrophy [1–4]. Natriuretic peptides have natriuretic and vasodilatory properties and beneficial actions on cardiac remodeling, so that the inverse relationship with BMI has been hypothesized to reflect a “natriuretic handicap”, with a reduced response to cardiac wall stress contributing to the initiation and progression of cardiovascular complications [5–7]. Consistently, in obese individuals NTproBNP plasma levels maintain significant prognostic information on the risk of developing heart failure, but equivalent accuracy is provided by lower thresholds values than in normal weight individuals [8–10].

Low circulating natriuretic peptides levels may results by abnormalities at various steps, including reduced cardiac secretion, reduced natriuretic type A receptor (NPRA) tissue signaling and increased systemic and tissue clearance by natriuretic peptide clearance receptor (NPRC) and neprilysin [11]. Moreover, genetic polymorphism in natriuretic peptide receptor sequences has also been shown, which might be involved in increased clearance and reduced circulating levels in homozygous C/C carriers [12]. Interestingly, prospective population-based studies have shown that low natriuretic peptides levels are associated with an increased risk of diabetes incidence [12–14] and an inverse relationship between natriuretic peptide and insulin sensitivity has also been found [15–17]. Further, in overweight and obese persons natriuretic peptides levels increase after either lifestyle intervention that reduced weight or bariatric surgery [18–22], although circulating levels remain lower than in persons with cardiovascular diseases.

Altogether, these findings suggest the alternative hypotheses that natriuretic peptides might act as either marker or risk factor of insulin-resistance mediated conditions, obesity, metabolic syndrome and diabetes [23]. However, data referring to the relationship between metabolic syndrome and natriuretic peptides are controversial, with studies showing both increased and reduced natriuretic peptides levels [24–27]. As regards to diabetes, increased plasma levels of NTproBNP have been identified as the strongest independent predictor of cardiovascular mortality even in people without pre-existing clinical CVD [28–30]. The paradox of both low and high levels of natriuretic peptides in different insulin-resistance mediated conditions such as obesity and diabetes, is intriguing. It has been suggested that an underlying dysregulation causing low natriuretic peptides might contribute to the increased metabolic and cardiovascular risk of insulin-resistant conditions, whereas the presence of subclinical CVD might be responsible of dragging NTproBNP values in the opposite direction, although they remain inadequate [31–33]. At present, however, studies comparing plasma levels of NTproBNP in population-based cohorts of both diabetic and non diabetic people are very limited [31], whereas clinic-based studies are potentially biased by both low numbers of examined people and limited generalizability of results. Therefore, the role of BMI on natriuretic peptide levels in people with and without diabetes has not been fully explored. In this paper we aimed to assess: (1) the cross-sectional relationship between BMI and NTproBNP in the full spectrum of metabolic diseases mediated by insulin-resistance, overweight/obesity, metabolic syndrome and diabetes; 2) the effect of CVD on NTproBNP levels in these conditions.

## Methods

The Casale Monferrato Study is an ongoing population-based study with extensive characterization of cardiovascular risk factors in both diabetic and non-diabetic people in a representative Italian population [28, 34]. In this report we have examined the relationship between NTproBNP and insulin-resistance mediated conditions, overweight/obesity, metabolic syndrome and type 2 diabetes.

The study-base were persons aged 45–74 years identified as part of two surveys of the population-based Casale Monferrato Study. The first survey recruited a diabetic cohort of 2315 people residents in 2000 in the town of Casale Monferrato, North-West of Italy (93,477 inhabitants) [28]. Out of them, centralized NTproBNP measurements were available in 1364 (59% of this cohort). The second survey recruited a non diabetic cohort from an age- and sex stratified sample of 3700 individuals, randomly identified in 2005–2006 through the files of the resident population of Casale Monferrato, after having excluded those with a previous diagnosis of diabetes or neoplastic diseases [34]. They received a letter and were further contacted by their general practitioner. Out of 3700 invited people, 2293 (62%) agreed to be interviewed and examined at the diabetes clinic. NTproBNP measurements were available in 1880 (82%). Therefore, present analyses included 3244 individuals aged 45–74 years, none of whom had heart failure, 1880 without diabetes and 1364 with diabetes.

The study protocol conforms to the ethical guidelines of the 1975 Declaration of Helsinki and the study protocol has been approved by the institutional ethical review committee. All patients were interviewed and examined, after having provided informed consent, at the local diabetes clinic of the S. Spirito Hospital, by trained investigators. Weight and height were measured with subjects not wearing shoes. Waist circumference was measured at the midpoint between the lower rib and the iliac crest and central obesity defined as values > 102 cm in men and > 88 cm in women. All laboratory determinations were centralized. Venous blood samples were collected after overnight fasting for determination of triglycerides, total cholesterol, high-density lipoprotein (HDL) cholesterol (enzymatic colorimetric method after precipitation with Mn^2+^). Low-density lipoprotein (LDL) cholesterol was calculated from the Friedewald’s formula for all persons in the cohort whose triglyceride values were < 4.48 mmol/l. The albumin excretion rate (AER) was calculated on the basis of the urinary albumin concentration measured in a single, timed, overnight urine sample by the nephelometric method (Behring Nephelometer Analyzer, Behring Institute, Marburg, Germany), after exclusion of urinary tract infection, congestive heart failure and other known causes of non diabetic renal disease. High-sensitivity CRP levels were measured using an immunoturbidimetric method (Roche Diagnostic). Serum NTproBNP levels were measured by a two-site sandwich electrochemiluminescence immunoassay (Elecsys proBNP II, Roche Diagnostic, Mannheim, Germany), using a Modular Analytics Evo analyzer with a E170 module (Roche). The intra-assay variation was below 3.0% and total CV ranges between 2.2 and 5.8% in low and high ranges of NTproBNP. Serum insulin was measured with radioimmunoassay in the non diabetic cohort only. The degree of insulin sensitivity was determined by the HOMA-IR, using the formula: fasting plasma glucose (mmol/l) times fasting serum insulin (mU/L) divided by 22.5. Blood pressure was measured with mercury sphygmomanometers to the nearest 2 mmHg, in the right arm at the start of examination, in sitting position, three consecutive times after an initial 5-min rest. Reported values are the average of second and third readings (phase 1 for systolic and phase 5 for diastolic pressure). Hypertension was defined as systolic blood pressure > 140 mmHg and/or diastolic blood pressure > 90 mmHg or treatment with antihypertensive drugs. Cardiovascular disease (CVD) was defined as physician diagnosed myocardial infarction, coronary artery bypass graft, stroke, arterial disease of lower limb or epiaortic trunks. The Rose questionnaire was also administered to allow the identification of people with symptoms suggestive of CVD (angina, IMA and arterial disease of lower limb). Coronary heart disease was also defined, on the basis of electrocardiographic abnormalities according to the Minnesota code, as probable (major Q and QS items, codes 1.1 and 1.2) or possible (minor Q and QS items, S-T/T items, codes 1.3, 4.1–4.4, 5.1–5.3). The diagnosis, however, had then to be confirmed by general practitioner/specialist according to standard examinations for these diseases. Smoking habit was classified into one of three categories: never smoker, ex-smoker if patient stopped smoking at least 1 month before the visit, and smoker. In the non diabetic cohort data on physical activity were assessed through a modified version of the International Physical Activity Questionnaire, including three question regarding activity at work, travel to and from places and recreational activities. The diagnosis of metabolic syndrome was defined according to the updated National Cholesterol Education Program’s Adult Treatment Panel III report.

### Statistical analyses

Variables distributed normally are presented as mean and standard deviation (SD), whereas variables with skewed distribution were analysed after natural logarithmic transformation (triglycerides, AER, creatinine, CRP, NTproBNP, HOMA-IR) and results presented as geometric means and interquartile range. Pearson correlations between NTproBNP and continuous variables were also performed. To explore the shape of the association between NTproBNP and BMI, we modeled NTproBNP as a restricted cubic spline in a multivariate linear model with BMI as dependent variable, adjusted for known risk factors and confounders (age, sex, hypertension, LDL-cholesterol, smoke, CRP, waist circumference, AER, creatinine, CVD). As in multiple linear regression we found a significant test of interaction between diabetes and NTproBNP (p < 0.0001), we performed separately analyses in diabetic and non diabetic people. In the whole cohort, we tested also the potential role of uric acid, HDL-cholesterol, triglycerides and that of a categorical variable with four levels determined by the combined effect of diabetes (yes/no) and overweight/obesity (BMI < 25 and ≥ 25 kg/m^2^). In non diabetic people, we tested the effect of HOMA-IR and physical activity and, in diabetic people, that of HbA1c, diabetes duration and diabetes treatment. All analyses were also performed after having excluded individuals with clinical CVD.

A logistic regression analysis was performed to assess the relationship between BMI (< 25 and ≥ 25 kg/m^2^, dependent variable) and quartiles of NTproBNP (< 6.40, 6.40–33.9, 34.0–89.9, > 89.9 pg/ml), independently of age, sex, diabetes, hypertension, LDL-cholesterol, waist circumference, smoke, CRP, AER, creatinine, CVD. As ORs in the upper quartiles of NTproBNP were similar, final models were performed using NTproBNP as a categorical dependent variable with two levels defined by its median value, to assess the independent ORs of overweight/obesity, diabetes and metabolic syndrome. All analyses were performed with Stata Release 10.0.

## Results

Overweight/obesity (BMI ≥ 25 kg/m^2^) was observed in 1118 (59.4%) of non-diabetic and 917 (67.2%) of diabetic subjects, respectively. Obesity (BMI ≥ 30 kg/m^2^) was observed in 345 (18.3%) of non diabetic and 469 (34.4%) of diabetic subjects. As shown in Table 1, significant differences among subgroups were evident, with overweight/obese people having the worst cardiovascular risk profile compared to normal weight people, irrespective of diabetes status. Among people with diabetes, NTproBNP levels were significantly lower in overweight/obese compared to normal weight subjects, but this difference was not observed in non diabetic subjects.

**Table 1:** Characteristics of the Casale Monferrato population-based cohort, by BMI and diabetes

	Non diabetes (n = 1880)			Type 2 diabetes (n = 1364)		
	BMI < 25 kg/m^2^ (n = 762)	BMI ≥ 25 kg/m^2^ (n = 1118)	p value	BMI < 25 kg/m^2^ (n = 447)	BMI ≥ 25 kg/m^2^ (n = 917)	p value
Age (years)	60.1 ± 8.3	61.4 ± 8.0	0.0007	64.7 ± 7.7	63.6 ± 8.0	0.01
Body mass index (kg/m^2^)	22.3 ± 2.0	28.9 ± 3.4	< 0.0001	23.8 ± 1.8	31.3 ± 4.6	< 0.0001
Waist circumference (cm)	80.9 ± 9.3	97.6 ± 9.7	< 0.0001	89.9 ± 10.5	103.2 ± 10.6	< 0.0001
Glucose (mmol/l)	4.99 ± 0.74	5.41 ± 1.18	< 0.0001	9.80 ± 3.31	9.89 ± 3.04	0.60
Total cholesterol (mmol/l)	5.69 ± 0.95	5.69 ± 1.02	0.89	5.60 ± 1.05	5.47 ± 1.03	0.08
LDL cholesterol (mmol/l)	3.29 ± 0.85	3.40 ± 0.90	0.007	3.39 ± 0.90	3.32 ± 0.88	0.24
HDL cholesterol (mmol/l)	1.88 ± 0.46	1.62 ± 0.40	< 0.0001	1.49 ± 0.41	1.34 ± 0.34	< 0.0001
Triglycerides (mmol/l)	1.01 (0.72–1.32)	1.32 (0.94–1.80)	< 0.0001	1.36 (0.94–1.91)	1.59 (1.10–2.15)	< 0.0001
Creatinine (µmol/l)	68.9 (60.1–78.7)	78.7 (64.5–84.9)	< 0.0001	84.9 (62.8–84.9)	72.5 (64.5–84.9)	0.007
CRP mg/l	0.11 (0.06–0.19)	0.22 (0.12–0.39)	< 0.0001	0.22 (0.10–0.48)	0.31 (0.14–0.67)	< 0.0001
Uric acid (µmol/l) mg/dl	269.44 ± 73.16	325.36 ± 80.3	< 0.0001	326.55 ± 146.32	333.68 ± 90.41	0.30
Systolic blood pressure (mmHg)	141.6 ± 20.4	150.1 ± 18.9	< 0.0001	143.3 ± 16.5	146.3 ± 16.1	0.001
Diastolic blood pressure (mmHg)	87.5 ± 10.5	93.0 ± 11.0	< 0.0001	81.2 ± 8.5	83.5 ± 8.2	< 0.0001
Hypertension (%)	479 (62.9%)	951 (85.1%)	< 0.0001	375 (84.6%)	830 (90.5%)	< 0.0001
People treated with						
Diuretics	83 (10.9%)	260 (23.3%)	< 0.001	119 (26.6%)	338 (36.7%)	< 0.001
ACE-inhibitors	82 (10.8%)	215 (19.2%)	< 0.001	153 (34.2%)	397 (43.3%)	< 0.001
ARBs	28 (2.3%)	83 (4.1%)	0.008	41 (9.2%)	107 (11.7%)	0.16
AER (µg/min)	3.4 (2.0–5.1)	4.6 (2.6–6.6)	< 0.0001	11.8 (4.2–23.9)	13.6 (4.5–30.5)	0.16
Smokers	141 (18.5%)	342 (30.6%)	< 0.0001	113 (25.9%)	303 (34.3%)	< 0.0001
CVD	23 (3.0%)	82 (7.3%)	< 0.001	90 (20.1%)	203 (22.1%)	0.40
NTproBNP (pg/ml)	14.6 (5–39.5)	13.4 (5–30.9)	0.13	77.3 (32–164)	62.4 (29–133)	0.006

Data are mean ± standard deviation and geometric means (interquartile range)

No significant correlation between NTproBNP and either BMI or waist circumference was found. In non diabetic people, NTproBNP values were positively correlated with age (r = 0.15, p < 0.0001) and AER (r = 0.05, p = 0.02) and negatively with HOMA-IR (r = −0.06, p = 0.02) and CRP (r = −0.07, p = 0.02). In diabetic people, we found significant positive correlations with age (r = 0.38, p < 0.0001), creatinine (r = 0.22, p < 0.001), CRP (r = 0.17, p < 0.001), AER (r = 0.15, p < 0.001) and systolic blood pressure (r = 0.08, p = 0.004).

We explored the existence of a non linear relationship between NTproBNP and BMI using cubic regression splines with knots at quintiles of distribution of NTproBNP, but no evidence was found of either non linear or curvilinear relationships, after multiple adjustments including diabetes. As interaction between NTproBNP and diabetes was significant (p < 0.001), multiple linear regression analyses were performed separately for diabetes and non diabetes. In non diabetic people, BMI was negatively associated with NTproBNP (β = −0.02 p = 0.03), independently of age, sex, creatinine, AER, CRP, smoke, physical activity, hypertension, LDL-cholesterol and CVD, but this association reverted to non significance after further adjustment for CRP, which was negatively related to NTproBNP (β = −0.15, p = < 0.0001). In diabetic people, waist circumference was negatively related to NTproBNP (β = −0.006, p = 0.04), and after further adjustment for CRP, β increased to −0.01 (p = 0.003), whereas CRP conferred a β value of 0.12 (p < 0.001). In this model, the categorical variable defining central obesity conferred a β of −0.24 (p = 0.001). HbA1c, diabetes duration and diabetes treatment did not contribute significantly to the model. Results were virtually identical limiting analyses to people without CVD.

Multiple linear regression analysis was then performed in the whole cohort to assess the individual and combined effect on NTproBNP values of a categorical variable with four levels defined by either individual or combined presence of overweight/obesity and diabetes, independently of other risk factors, obtaining the following results: non diabetes and overweight/obesity, β = −0.12 (p = 0.03); diabetes and normal weight, β = 1.28, (p < 0.0001); diabetes and overweight/obesity β = 1.03 (p < 0.0001). After further adjustment for CRP, the β value of the category defined by non diabetes and overweight/obesity reverted to non statistical significance (p = 0.06).

In a logistic regression model examining the relationship between overweight/obesity (dependent variable) and quartiles of NTproBNP, independently of diabetes and other confounders, we obtained the following ORs: 0.84 (0.64–1.11), 0.68 (0.50–0.93) and 0.53 (0.37–0.77). Therefore, final analyses were performed using NTproBNP as a dependent categorical variable with two levels defined by its median value (34.0 pg/ml) assessing the independent ORs of having high NTproBNP by metabolic categories (Table 2). Model 1 explored the individual effect of BMI and diabetes. As compared to normal-weight individuals, those with a BMI ≥ 25 kg/m^2^ had an adjusted OR of 0.70 (95% CI 0.56–0.87), independently of diabetes, whereas individuals with diabetes showed a 2.6-fold increase in the adjusted OR compared to non diabetic subjects, independently of BMI. We then examined the effect of diabetes combined with overweight/obesity (Model 2), and that of the metabolic syndrome (Model 3). In people with overweight/obesity only, the OR of having NTproBNP levels above the median value was significantly lower than in normal weight subjects (Model 2). The presence of diabetes either alone or combined with overweight/obesity (Model 2) or metabolic syndrome (Model 3) greatly enhanced the OR of having NTproBNP levels above the median value, while the presence of metabolic syndrome alone had a more modest effect (OR 1.54, 1.18–2.01). All previous results were virtually identical after the exclusion of subjects with CVD.

**Table 2:** Adjusted odds ratios (ORs) of having NTproBNP levels above median value (34 pg/ml) in people aged 45–74 years in the population-based Casale Monferrato Study

	Adjusted OR* (95% CI)
Model 1	
BMI < 25 kg/m^2^	1.00
BMI ≥ 25 kg/m^2^	0.70 (0.56–0.87)
Non diabetes	1.00
Diabetes	2.60 (2.37–2.87)
People without CVD	
BMI < 25 kg/m^2^	1.00
BMI ≥ 25 kg/m^2^	0.67 (0.53–0.86)
Non diabetes	1.00
Diabetes	2.47 (2.23–2.74)
Model 2	
Non diabetes, BMI < 25 kg/m^2^	1.00
Non diabetes, BMI ≥ 25 kg/m^2^	0.63 (0.48–0.82)
Diabetes, BMI < 25 kg/m^2^	5.44 (3.98–7.44)
Diabetes, BMI ≥ 25 kg/m^2^	4.42 (3.25–6.01)
P for trend	< 0.0001
People without CVD	
BMI < 25 kg/m^2^	1.00
BMI ≥ 25 kg/m^2^	0.64 (0.48–0.88)
Non diabetes	5.47 (3.93–7.62)
Diabetes	4.13 (2.99–5.72)
P for trend	< 0.0001
Model 3	
Neither Metabolic syndrome nor diabetes	1.00
Metabolic syndrome	1.67 (1.25–2.22)
Diabetes	7.45 (5.54–10.01)
Metabolic syndrome + diabetes	7.56 (5.84–9.78)
P for trend	< 0.0001
People without CVD	
Neither Metabolic syndrome nor diabetes	1.00
Metabolic syndrome	1.99 (1.48–2.69)
Diabetes	7.49 (5.47–10.3)
Metabolic syndrome + diabetes	7.52 (5.70–9.92)
P for trend	< 0.0001

* ORs are adjusted age, sex, waist circumference, plasma creatinine, hypertension, LDL-cholesterol, smoke, CRP, AER and CVD

Finally, we performed a separate analysis in the non-diabetic cohort to examine the effect of HOMA-IR values above median value (≥ 2.0) on NTproBNP (Table 3), after having excluded individuals with CVD. As shown in Model 1, BMI ≥ 25 kg/m^2^ and HOMA-IR ≥ 2.0 decreased to a similar extent the ORs of having NTproBNP above the median value (BMI OR 0.70, 95% CI 0.55–0.89, HOMA-IR OR 0.68, 0.54–0.87). However, the association between overweight/obesity and NTproBNP was marginally not significant after the inclusion into the model of HOMA-IR (Model 2). CRP was negatively associated with NTproBNP and this effect was evident even in the fully adjusted model (Model 4).

**Table 3:** Adjusted odds ratios (ORs) of having NTproBNP levels above median value (34 pg/ml) in non diabetic people aged 45–74 years without CVD, in the population-based Casale Monferrato Study

	Adjusted OR* (95% CI)			
	Model 1	Model 2	Model 3	Model 4
BMI ≥ 25 kg/m^2^	0.70 (0.55–0.89)	0.78 (0.61–1.01)		0.83 (0.64–1.08)
HOMA-IR ≥ 2.04	0.68 (0.54–0.87)	0.74 (0.58–0.96)	0.74 (0.58–0.94)	0.78 (0.61–1.01)
CRP > 3 mg/l	0.59 (0.45–0.78)		0.63 (0.47–0.83)	0.65 (0.49–0.86)

Variables were included separately in model 1 and simultaneously in all other models

* ORs are adjusted age, sex, waist circumference, physical activity, plasma creatinine, hypertension, LDL-cholesterol, smoke, CRP, and AER

## Discussion

This study assessed the cross-sectional relationship between NTproBNP and insulin-resistance associated conditions—overweight/obesity, metabolic syndrome, and diabetes—in the large population-based cohort of diabetic and non-diabetic individuals from the Casale Monferrato Study. We provided evidence that overweight/obese subjects had a 30% lower OR of having NTproBNP levels above median values compared to normal weight individuals (Table 2, Model 1). This finding, which was independent of the presence of diabetes and other confounders, such as chronic renal failure, was evident even after having excluded people with clinical CVD. Therefore, we provide further evidence supporting the hypothesis of a “natriuretic handicap” in overweight/obese individuals, which is evident even in those with diabetes, although higher prevalence of subclinical CVD would force natriuretic peptides levels in the opposite direction [5–7]. Our findings are in agreement with previous epidemiological studies that consistently showed a negative association between BMI and NTproBNP levels in non diabetes [1–4, 35], whereas data including diabetes are more limited [31–33]. The cause of the relative BNP/NTproBNP deficiency in obesity (natriuretic handicap) is poorly understood, and several mechanisms have been suggested, including reduced BNP synthesis/release from the heart and increased peripheral degradation [5–7]. Natriuretic peptides are cleared and degradated by neutral endopeptidase neprilysin and NPRC [23]. Interestingly, insulin has been observed to induce NPRC expression in human adipocytes [36, 37], and this finding might link conditions associated with hyperinsulinemia, such as obesity and insulin-resistance. Moreover, neprilysin, the natriuretic peptides degrading endopeptidase, is expressed at increased levels in obesity [38]. On the other hand, there is also emerging evidence that natriuretic peptides control metabolic processes by enhancing lipolysis and energy expenditure, acting at mitochondrial level [39]. Therefore, low NTproBNP levels in overweight/obesity may be not only a consequence, but also a cause of obesity. Indeed, low levels of NTproBNP might lead to reduced lipolysis and excessive weight gain, which may be one of the biological alterations that contribute to the development of metabolic syndrome [11]. From a clinical point of view, however, it is relevant to notice that even in obese individuals NTproBNP levels provide significant prognostic information of risk of developing heart failure [8, 9]. Indeed, lower cut-off values in obese individuals have been found to provide equivalent accuracy to values applied for non obese individuals, and this further support the “natriuretic handicap” hypothesis, with reduced response to cardiac wall stress associated with obesity [8].

### Insulin resistance, CRP and NTproBNP

In our study the inverse association between BMI and NTproBNP, observed in non-diabetic patients without CVD, was no longer significant after the inclusion of insulin resistance (HOMA-IR) into the model (Table 3, Model 2), confirming that insulin-resistance is important in linking overweight/obesity to reduced NTproBNP levels. This finding has been previously observed in two large community-based studies, the Framingham Heart Study and the Malmo Diet and Cancer study, showing that the inverse relationship between obesity and NTproBNP was attenuated after adjustment for HOMA-IR [15]. Moreover, this relationship was evident not only in obese but also in non obese individuals [15]. Even in the elderly, the Cardiovascular Health Study showed that lower NTproBNP levels were associated with higher insulin resistance in individuals without heart failure, CHD and chronic kidney disease [16]. In the intervention Diabetes Prevention Program study, circulating NTproBNP was associated with a measure of insulin sensitivity before and during preventive interventions regardless of whether a participant was treated with placebo, intensive lifestyle intervention or metformin [17]. Statin treatment has also been reported to induce insulin resistance and, in line with previous findings, a small clinic-based study found that the inverse relationship between NTproBNP and HOMA-IR was limited to individuals treated with statin [40]. Altogether, these findings provides further support to the current hypothesis that NTproBNP acts as marker of insulin sensitivity, independently of BMI. However, only prospective studies assessing whether directly altering BNP concentrations influence metabolic risk would allow to disentangle the role of natriuretic peptides as marker or determinant of insulin sensitivity.

As a chronic low-grade inflammation triggered by adipocyte hypertrophy is considered an important mechanism coupling an unhealthy excess body fat to insulin-resistance [41], we have also explored the potential role of CRP, a marker of chronic inflammation. In non-diabetic patients, CRP values were inversely correlated with NTproBNP levels and this inverse relationship was confirmed in logistic regression, independently of other risk factors. Importantly, the inverse association between NTproBNP and both BMI and insulin resistance was no longer significant after the inclusion of CRP into the logistic regression model (Table 3, Model 4) consistently with the hypothesis that obesity-related insulin resistance is, at least in part, a chronic inflammatory disease initiated in adipose tissue [42]. Our finding of a negative relationship between CRP and NTproBNP in non diabetic people likely reflects the role of CRP as marker of other abnormalities directly affecting circulating NTproBNP and related to adiposity. We did not examine the roles of adiposite generated cytokines, such as tumor necrosis factor-α, interleukin-1 (IL-1) and IL-6, promoting the release of CRP, but it is known that BNP is upregulated at the transcriptional and translational levels by pro-inflammatory cytokines in cardiac myocytes [43]. Moreover, a recent study found a significant positive correlation between the plasma BNP and serum CRP levels in cancer patients as well as cancer model mice without overt heart failure, linking also cancer-related chronic inflammation and natriuretic peptides [44]. In the MESA Study, including 5597 individuals (12% with diabetes), IL-6 was positively associated with NTproBNP throughout its whole range of values [31]. In our study, CRP values were twofold higher in individuals with diabetes (42% of the overall cohort) than in those without diabetes and a positive correlation between CRP and NTproBNP was also found in diabetes only, consistently with higher prevalence of CVD in diabetes, and increased release of cytokines and CRP production [43, 45]. In line with these findings, in the EURODIAB Prospective Complications Study we previously showed with multivariate analyses that increased NTproBNP values in type 1 diabetes complications were explained by increased TNF-α levels [46].

### Diabetes and NTproBNP

People with diabetes had a 2.6-fold increased OR of having NTproBNP values above median levels as compared to people without diabetes, independently of BMI, confounders and risk factors (Table 2, Model 1). Because diabetes and BMI exert opposite effects on NTproBNP, the independent association between diabetes and NTproBNP was much stronger in normal-weight subjects (Table 2, Model 2) whereas people with overweight/obesity had 30% higher likelihood of having lower levels. Moreover, in multiple linear regression central obesity was negatively associated with NTproBNP levels, independently of other confounders. Therefore, the effect of diabetes prevailed on that of BMI and NTproBNP values were reduced in people with both diabetes and overweight/obesity, pointing out the existence of a likely natriuretic handicap even in diabetic subjects [23]. Consistently, different BNP threshold have been identified in diabetic compared to non diabetic patients to maintain equivalent accuracy in screening for stage B heart failure [47].

In our study the issue of a direct relationship between diabetes and NTproBNP is clinically relevant as NTproBNP is a potent marker and predictor of vascular complications [28–30, 46]. This relationship was independent of CVD, AER, serum creatinine, and hypertension. Therefore, the rise in NTproBNP levels was not explained by the presence of cardiovascular and renal chronic complications of diabetes and/or by hemodynamic changes that are known to enhance NTproBNP levels. Diabetes may induce alterations in natriuretic peptides processing and/or clearance resulting in enhanced NTproBNP circulating levels [39]. Alternatively, subclinical pathological processes occurring in the cardiovascular system of diabetic patients may enhance BNP/NTproBNP levels. Consistent with this hypothesis, in the Multi-Ethnic Study of Atherosclerosis when subclinical CVD develops, the inverse relationship between NTproBNP and BMI was completely lost and NTproBNP levels rise, likely as the result of subclinical CVD [31]. This would also explain why NTproBNP is a strong predictor of diabetes cardiovascular complications [33]. In the whole cohort of the Casale Monferrato Study we found no evidence of either non linear or curvilinear relationship between NTproBNP and other variables as reported in the MESA study [31]. However, we found a strong modification effect of diabetes and this finding might be related to the higher frequency of individuals with diabetes examined in our study (42%) compared to the MESA Study (12%). Potential mechanisms of the rise in NTproBNP circulating levels in this setting are hypoxia, enhanced myocardial wall strain, and inflammatory cytokines as they have been shown to increase BNP production [23]. In this regard it is noteworthy that in our study the correlation between CRP and NTproBNP, that was negative in obese/overweight non diabetic individuals, became positive in those with diabetes, even in those without pre-existing CVD, suggesting the effect of subclinical CVD on both CRP and NTproBNP plasma levels.

### Metabolic syndrome and NTproBNP

The presence of metabolic syndrome increased the OR of having NTproBNP values above median levels (Table 3, Model 3) and there was a significant trend of increasing OR through classes of metabolic abnormalities, with the lowest OR observed in people who did not have either diabetes or metabolic syndrome and the highest OR in people having both. Previous data on the relationship between NTproBNP and the metabolic syndrome have been conflicting, with studies showing either lower or similar natriuretic peptides values compared to people without the metabolic syndrome [25, 26]. This is likely due to differences among examined populations in the relative frequencies of metabolic syndrome components with opposite effects on NTproBNP, such as obesity and hypertension. Consistently, in a case-cohort analysis of the Casale Monferrato Study we provided evidence that, compared to people without any component of the metabolic syndrome, those in the uncomplicated phase of the syndrome, who had neither CVD/chonic renal failure nor diabetes, had yet increased NTproBNP values, even if they were normotensive [27].

### Limitation and strength

There are certain limitations to our study. First, it is a cross-sectional study and this restricts our ability to assess temporal relationships and to identify underlying causal biological mechanisms. Second, although people with acute heart failure were excluded, no data on structural and functional cardiac abnormalities were available and we could not adjust data either for echocardiographic variables such as left ventricular mass and left atrial size or for subclinical CVD. Third, physical activity level was examined in the non diabetic cohort only. Finally, we measured NTproBNP plasma levels only as a marker of BNP. As circulating levels of proBNP 1-108 are not detected by conventional BNP1-32 and NTproBNP assays, we cannot exclude the hypothesis of impaired peripheral processing to mature BNP 1-32 [23].

The strength of this analysis is the large sample size and broad distribution of age, increasing precision and generalizability. Indeed, our study included two large population-based cohorts of people living in the same area and examined with standardized methods, with similar numbers of recruited people with and without type 2 diabetes. This allowed us to perform comparisons among different groups of metabolic abnormalities.

## Conclusions

Altogether, these findings, although derived from a cross-sectional study, provides further epidemiological evidence towards the current hypothesis that natriuretic peptides are implicated in metabolic processes, with lower circulating levels in insulin-resistance non diabetic people and higher values in the upper spectrum of metabolic abnormalities, diabetes with the metabolic syndrome. Moreover, diabetic overweight individuals have higher but inadequately increased levels of NTproBNP compared to normal weight diabetic individuals, suggesting an underlying natriuretic handicap. These results were irrespective of presence/absence of CVD, which by itself increases natriuretic peptides levels. However, further studies are required to test the possibility that new intervention strategies targeting the natriuretic peptides system may be effective in lowering not only blood pressure and sodium retention, but also body weight and the risk of diabetes.

## Abbreviations

CVD: cardiovascular diseases
CHD: coronary heart disease
BMI: body mass index
OR: odds ratio
AER: albumin excretion rate
CRP: C-reactive protein
